# When expertise recalibrates perception: skill-dependent dissociation between perceived effort and objective force in calligraphy

**DOI:** 10.3389/fpsyg.2026.1775281

**Published:** 2026-03-16

**Authors:** Lujia Hao, Pengkai Chen

**Affiliations:** 1 College of Education, Bangkok Thonburi University, Bangkok, Thailand; 2 College of Teachers Education, Zhejiang Normal University, Jinhua, China

**Keywords:** aesthetic judgment, embodied cognition, force perception, motor expertise, perceptual dissociation, skill acquisition

## Abstract

**Introduction:**

Expertise is traditionally associated with enhanced perceptual accuracy, yet we demonstrate a paradoxical pattern in skilled motor performance: greater expertise corresponds to systematic functional recalibration of effort perception relative to objective force. This study examined how calligraphy skill development alters perceived effort and its consequences for aesthetic judgment, from an embodied cognition perspective.

**Methods:**

Sixty calligraphy learners (20 novices, 20 intermediates, 20 experts) wrote five standard Chinese characters while pressure sensors and surface electromyography simultaneously recorded pen-tip pressure, upper limb muscle activation, and subjective effort ratings. Professional judges evaluated the aesthetic quality of all works.

**Results:**

Results revealed systematic skill-dependent perceptual recalibration: experts’ perceived effort deviated from actual pressure by 60% (recalibration index = 0.347), 4.5 times greater than novices’ 15% deviation (0.078), with intermediates showing 40% deviation (0.201). This functional recalibration was strongly associated with muscle synergy reorganization (*r* = 0.74): experts developed a “proximal-dominant, distal-refined” activation pattern, with increased shoulder and arm muscle engagement despite reduced pen-tip pressure. Critically, in predicting aesthetic ratings, perceived effort was a powerful predictor (*β* = 0.58, *p* < 0.001) while actual pressure showed no predictive value (*β* = 0.02, *p* = 0.658). Mediation analysis revealed that perceptual recalibration accounted for 63.2% of skill level’s effect on aesthetic judgment.

**Discussion:**

These findings suggest that perception in skilled performance undergoes functional recalibration to optimize task performance, reflecting an adaptive reorganization shaped by embodied experience rather than veridical representation of physical force. The systematic perceptual recalibration with expertise reveals that expert perception prioritizes task-relevant functional information over veridical physical accuracy.

## 1 Introduction

An intriguing phenomenon exists in the practice and appreciation of Chinese calligraphy: expert calligraphers’ subjective experience of effort during writing systematically diverges from objectively measured pen-tip pressure. This divergence between perceived effort and objective force is not an isolated phenomenon but may represent a functional adaptation. Skill development research indicates that experts’ perceptual experiences may undergo systematic reconstruction as skills are acquired (Castro-Alonso et al., 2024). In the motor skill domain, researchers have found that the sensorimotor system undergoes adaptive changes with professional training (Hosoda et al., 2020; Park and Caldwell, 2022; Yadav et al., 2025), but how these changes affect perceived effort, particularly in fine motor skills such as calligraphy, remains underexplored. This study aimed to investigate the neuromuscular mechanisms underlying this functional perceptual recalibration and its influence on aesthetic judgment from an embodied cognition perspective.

Embodied cognition theory provides an important theoretical framework for understanding this phenomenon (Barsalou, 2008; Wilson, 2002). Recent reviews suggest that cognitive processes are deeply rooted in the body’s sensorimotor experiences, with bodily states and action experiences shaping our perception and understanding of the world (Castro-Alonso et al., 2024; Körner et al., 2023). During skill acquisition, repeated bodily practice not only changes how actions are executed but also functionally recalibrates perceived effort to align with task-relevant motor representations (Hommel et al., 2001; Tsay et al., 2022). Research on the mirror neuron system further indicates that observing and executing actions activate similar neural circuits (Rizzolatti and Craighero, 2004), allowing motor experience to influence our understanding of others’ actions (Kirsch et al., 2016). Particularly in professional domains such as dance and musical performance, studies have found that experts’ observational experiences are significantly modulated by their own motor skills (Darda and Cross, 2022). These findings suggest that calligraphers’ unique perception of force may stem from embodied motor representations formed through long-term training.

The development of calligraphy skills is accompanied by the formation of complex muscle coordination patterns. Neuroscience research indicates that skilled motor control is not achieved through independent control of each muscle, but rather through organizing muscle synergies—functional neural modules that coordinate the co-activation of multiple muscles—to simplify the control problem (d'Avella et al., 2003). During skill learning, the structure and activation patterns of muscle synergies undergo systematic changes to adapt to task demands (Park and Caldwell, 2022). In the calligraphy domain, research suggests that calligraphy practice involves fine muscle control and coordination processes, with integration of upper limb visual perception, proprioception, and motor systems being crucial for achieving fluid writing movements (Yue et al., 2023). Muscle synergy analysis methods have been widely applied in upper limb movement assessment (Zhao et al., 2021). As skill level increases, calligraphers may develop more efficient muscle synergy patterns, and this neuromuscular reorganization may be key to understanding changes in perceived effort.

From an embodied aesthetics perspective, viewers’ aesthetic experience of artworks is not a purely visual process but involves bodily motor simulation and emotional resonance (Freedberg and Gallese, 2007). Research indicates that when we observe brushstrokes in paintings or forms in sculptures, the brain automatically simulates the actions required to produce these traces, and this embodied simulation constitutes an important foundation for aesthetic experience (Calvo-Merino et al., 2008; Kirsch et al., 2016). Neuroimaging studies further confirm that observing artworks activates the observer’s motor cortex, and this activation is closely related to the observer’s own motor experience (Umiltà et al., 2012). Notably, the intensity of embodied simulation is modulated by individual motor skill level—experts show stronger neural activation and deeper emotional engagement when viewing actions related to their expertise (Darda and Cross, 2022). In calligraphy appreciation, this suggests that viewers may sense the force and rhythm of works by simulating writing movements, and this simulated experience may be based not on objective physical force but on the calligrapher’s subjective perception of effort during creation. Thus, calligraphers’ perceptual recalibration may not only affect their own creative experience but also influence viewers’ aesthetic judgment through embodied simulation mechanisms.

Although the above research provides a theoretical foundation for understanding the relationship between force perception and skill development, several important research gaps remain. First, regarding whether experts exhibit systematic functional recalibration between actual force and perceived force, and the magnitude and characteristics of this recalibration, quantitative empirical data are currently lacking. Second, although both muscle synergy theory and embodied cognition theory emphasize the role of the body in cognition (Park and Caldwell, 2022), how these two theoretical frameworks are interrelated in specific skill contexts remains unclear. Third, in the calligraphy domain, existing research has explored motor control characteristics and muscle coordination in calligraphy practice (Yue et al., 2023), but these studies primarily focus on the rehabilitative effects and psychological impacts of calligraphy practice; systematic research on the embodied mechanisms of force perception during calligraphers’ creative process and its relationship with muscle synergy patterns remains lacking. Finally, how the divergence between perceived force and actual force influences aesthetic judgment, and whether this influence varies with viewers’ skill level, remain questions to be explored. Skill acquisition research indicates that individuals at different expertise levels exhibit qualitative differences in perceiving and executing motor tasks (Araujo et al., 2023), but how these differences manifest in aesthetic judgment remains unclear.

Therefore, this study proposed three research questions: (1) Do calligraphers at different skill levels exhibit systematic functional recalibration between perceived effort and actual pressure during writing? What is the relationship between this bias and skill level? (2) Is perceptual recalibration associated with specific muscle synergy patterns? How do calligraphers at different skill levels differ in muscle coordination strategies? (3) In aesthetic judgment, which better predicts aesthetic ratings: perceived effort or actual pressure? Does viewers’ skill level moderate this relationship? Through multimodal measurements including pressure sensors, electromyography, and aesthetic ratings, this study aimed to reveal the neuromuscular mechanisms underlying the perceived effort paradox and explore its influence on aesthetic judgment in calligraphy, providing empirical support for the application of embodied cognition theory in the artistic domain.

## 2 Methods

### 2.1 Participants

Sixty calligraphy learners were recruited through convenience sampling and divided into three groups based on learning duration and practice frequency: novice group (*N* = 20, learning duration < 6 months, practice < 2 h/week), intermediate group (*N* = 20, learning duration 1–3 years, practice 3–5 h/week), and expert group (*N* = 20, learning duration > 5 years, practice > 5 h/week with competition or exhibition experience). This classification scheme is consistent with neuroimaging studies of Chinese calligraphy expertise, which have established ≥5 years of formal training as a validated threshold for expert-level performance (Chen et al., 2017; Wang et al., 2019). The three-level classification allows for examination of skill development across the learning trajectory from initial acquisition through intermediate proficiency to expert automaticity. Sample size was determined to provide adequate statistical power (>0.80) for detecting large between-group effects (*d* ≥ 0.8), which are typical in expert-novice comparisons of domain-specific motor skills, and is consistent with sample sizes used in comparable neuroimaging and motor expertise research (Szucs and Ioannidis, 2020).

Novice group participants were primarily recruited from university calligraphy elective courses and beginner-level training classes, intermediate group from calligraphy clubs and advanced training classes, and expert group through calligraphy associations and art academy graduate student populations. All participants were right-handed, had normal or corrected-to-normal vision, and had no history of neurological disorders or motor impairments. All participants provided written informed consent before the experiment.

As shown in Table 1, the three groups showed no significant differences in age, gender ratio, or years of education (*p* > 0.05), with significant differences only in calligraphy learning duration and weekly practice hours, meeting the skill level grouping criteria.

**Table 1:** Demographic information of participants.

Variable	Novice (*N* = 20)	Intermediate (*N* = 20)	Expert (*N* = 20)	*F*/*χ*^2^	*p*
Age (years), *M* ± SD	23.4 ± 3.2	24.1 ± 4.5	25.8 ± 5.1	1.45	0.243
Gender (M/F)	9/11	11/9	10/10	0.31^a^	0.856
Education (years), *M* ± SD	15.2 ± 2.1	15.8 ± 2.4	16.1 ± 2.8	0.67	0.516
Learning duration (months), *M* ± SD	3.2 ± 1.8	26.4 ± 8.7	78.6 ± 24.3	156.78^***^	<0.001
Weekly practice (h), *M* ± SD	1.3 ± 0.6	4.1 ± 0.9	6.8 ± 1.5	124.56^***^	<0.001

^a^Chi-square test was used for gender distribution; ^***^p < 0.001.

### 2.2 Apparatus and materials

A thin-film pressure sensor array (Tekscan Model 5,101, sampling rate 100 Hz, accuracy ±0.5%) was placed beneath the writing surface to record vertical pressure applied by the pen tip in real time (Schwellnus et al., 2013). A surface electromyography (EMG) system (Delsys Trigno Wireless, 4 channels) measured electrical activity of the forearm flexor group (electrode placed on the palmar side of the forearm, primarily collecting signals from flexor carpi radialis and palmaris longus), forearm extensor group (electrode placed on the dorsal side of the forearm, primarily collecting signals from extensor carpi radialis and extensor carpi ulnaris), biceps brachii, and anterior deltoid, with a sampling rate of 2,000 Hz and bandpass filter of 20–450 Hz (Zaman and Stegemöller, 2018). The two systems were time-synchronized via hardware trigger (error < 1 ms). A video recording system with side and overhead views was used for subsequent movement analysis and participant playback.

Writing materials consisted of standardized medium-sized dual-hair brushes (brush length 4.5 cm, diameter 0.8 cm), six-foot quarter-size cooked Xuan paper (45 × 34 cm), and commercially bottled ink diluted to standard concentration. Five commonly used characters—"永 (yǒng),” “之 (zhī),” “一 (yī),” “大 (dà),” and “天 (tiān)”—were selected as writing tasks. These characters contain typical lifting and pressing movements with 5, 3, 1, 3, and 4 strokes respectively, totaling 16 basic strokes, suitable for experimental control and data collection.

### 2.3 Procedure

The experiment was conducted in a quiet laboratory with temperature controlled at 22–24 °C and humidity at 50–60%. Upon arrival, participants first completed a basic information questionnaire and calligraphy learning history survey, followed by 5 min of warm-up writing to adapt to the environment and experimental equipment. The formal experiment included two tasks with a total duration of approximately 25 min.

Task 1 involved writing and immediate effort rating. Participants sat at a standard desk and wrote five standard characters using a natural pen grip, with each character written three times, yielding 15 character samples. The five characters comprised 16 basic strokes, generating pressure and EMG data for 48 strokes per participant (16 strokes × 3 repetitions). During writing, the pressure sensor and EMG system simultaneously recorded data. After completing each stroke, a research assistant immediately asked verbally: “How much effort did you feel you exerted when writing this stroke?” Participants responded using a 0–10 scale (0 = no effort at all, 10 = maximum possible effort). Perceived effort was defined as the conscious sensation of how hard, heavy, and strenuous the writing task felt—that is, how hard participants felt they were trying to produce the stroke (Marcora, 2010; Pageaux, 2016). To ensure consistent understanding, participants received standardized instruction training before the experiment, clarifying that “effort” referred to how hard they felt they were trying during writing (rather than the visual effect of the stroke), and understanding of task requirements was confirmed through 2–3 practice trials. To assess rating reliability, 10 randomly selected participants repeated the task 1 week later, with test–retest reliability ICC = 0.78, indicating stable and reliable effort ratings.

Task 2 involved aesthetic rating. Participants rated each of their written character samples (15 characters total) on “force aesthetics” (0–10 scale, 0 = no force aesthetics at all, 10 = extremely strong force aesthetics). This task was conducted 15 min after writing completion, during which participants rested and completed a fatigue questionnaire. Additionally, 10 graduate students majoring in calligraphy were recruited as independent raters to blindly evaluate all participants’ works, with each work rated by 3 raters and the average taken as the objective aesthetic rating. Inter-rater reliability was ICC = 0.82, indicating reliable ratings. Ten randomly selected participants repeated the self-rating task 1 week later, with test–retest reliability ICC = 0.81.

### 2.4 Data processing

Pressure data processing included: extracting peak pressure, average pressure, and pressure standard deviation for each stroke during writing, and removing obvious noise signals (such as impact force at pen lift and contact noise before pen placement). This study used average pressure as the operational indicator of “actual pressure” because it more stably reflects the pressure level throughout the stroke writing process, avoiding the problem of peak pressure being susceptible to momentary disturbances.

EMG data processing included: calculating root mean square (RMS) values after full-wave rectification, normalizing as a percentage of maximum voluntary contraction (MVC), and calculating the average activation level of four muscles during each stroke. The muscle synergy index was defined as the sum of normalized RMS for proximal muscles (biceps brachii + deltoid) divided by the sum of normalized RMS for distal muscles (forearm flexors + extensors), reflecting the degree of effort pattern shift from distal to proximal. EMG data processing included: calculating root mean square (RMS) values after full-wave rectification, normalizing as a percentage of maximum voluntary contraction (MVC), and calculating the average activation level of four muscles during each stroke. The muscle synergy index was defined as the sum of normalized RMS for proximal muscles (biceps brachii + deltoid) divided by the sum of normalized RMS for distal muscles (forearm flexors + extensors), reflecting the degree of effort pattern shift from distal to proximal.

It should be noted that this synergy index represents a simplified operationalization rather than synergy extraction based on established algorithms such as non-negative matrix factorization (NNMF). We adopted this ratio-based measure for several reasons. First, as an exploratory study examining the embodied basis of perceptual recalibration, this index directly captures the key proximal-to-distal shift in effort distribution that is theoretically relevant to our research questions. Second, NNMF-based synergy extraction typically requires larger sample sizes and more extensive muscle recordings (typically 8–16 channels) to achieve stable decomposition (d'Avella et al., 2003), whereas our 4-channel EMG configuration was optimized for the specific proximal-distal contrast of interest. Third, similar ratio-based indices have been used in prior motor control research as gross indicators of coordination patterns (Park and Caldwell, 2022). Nevertheless, we acknowledge that this measure may conflate overall effort magnitude with coordination strategy, and that ratio measures are statistically sensitive to denominator variance. Future research should employ NNMF or other synergy extraction methods with more comprehensive muscle recordings to provide more rigorous characterization of coordination reorganization during skill acquisition.

The perceptual recalibration index was calculated using a whole-sample normalization method to ensure comparability across participants. Since actual pressure (unit: Newtons) and subjective effort ratings (0–10 scale) are not comparable in terms of dimensions, we normalized all participants’ actual average pressure and effort ratings separately to a 0–1 range using the maximum and minimum values of the entire sample. The normalization formula was: (original value − sample minimum)/(sample maximum − sample minimum). The recalibration index was then calculated as: normalized perceived effort—normalized actual pressure. This method eliminated dimensional differences while preserving absolute differences between participants. A positive value indicated that perceived effort for that stroke was higher than actual pressure (relative to the entire sample distribution), reflecting upward perceptual recalibration, while a negative value reflected downward recalibration.

Data aggregation employed different levels across analyses. Stroke-level analyses used raw data for each stroke (48 strokes per participant, total *n* = 2,880). Participant-level analyses averaged the 48 stroke data points for each participant, yielding participant-level summary indicators (*n* = 60). Character-level analyses calculated the average of strokes contained in each character, including average recalibration index, average actual pressure, and average perceived effort (15 characters per participant, total *n* = 900). Data aggregation employed different levels across analyses. Stroke-level analyses used raw data for each stroke (48 strokes per participant, total *n* = 2,880). Participant-level analyses averaged the 48 stroke data points for each participant, yielding participant-level summary indicators (*n* = 60). Character-level analyses calculated the average of strokes contained in each character, including average recalibration index, average actual pressure, and average perceived effort (15 characters per participant, total *n* = 900). Specifically, for character-level aggregation, all values for strokes within each character were averaged using arithmetic mean. For example, the character “永” contains 5 strokes, so its character-level recalibration index was calculated as the mean of the recalibration indices of these 5 strokes.

### 2.5 Statistical analyses

Statistical analyses employed two approaches depending on data structure. For participant-level variables (single value per participant, *n* = 60), one-way analysis of variance (ANOVA) was used to compare means across the three skill groups (novice, intermediate, expert). For stroke-level and character-level variables (nested data with multiple observations per participant), multilevel linear modeling was employed to account for within-participant correlation.

For ANOVA analyses, the following statistics were reported: F-statistic with degrees of freedom (between-groups df, within-groups df), *p*-value, and effect size (partial *η*^2^). Post-hoc pairwise comparisons were conducted using Tukey’s HSD method when the omnibus *F*-test was significant (*α* = 0.05). For multilevel linear modeling analyses, fixed effects were tested using *t*-tests, and model fit was assessed using conditional *R*^2^.

Multilevel linear modeling. For nested data structures (strokes nested within participants, or characters nested within participants), multilevel linear modeling (hierarchical linear modeling) was employed. The basic model included two levels: Level 1 for observation units (strokes or characters), and Level 2 for participants. The Level 1 model was: $Y_{\mathrm{ij}}=\beta_{0j}+\varepsilon_{\mathrm{ij}}$ , where *Y* represents the dependent variable (such as deviation index, EMG indicators, or aesthetic rating), *i* represents the observation unit, *j* represents the participant, and *ε_ij_* is the residual. The Level 2 model was: $\beta_{0j}=\gamma_{00}+\gamma_{01}(\text{skill level}j)+u0j$ , where skill level was a categorical variable with the novice group as the reference group, *γ*_01_ represents the fixed effect of skill level, and *u*₀*j* represents the random effect at the participant level.

Nested model comparison. To test the independent contribution of muscle synergy index to deviation index while controlling for skill level, nested models were compared. The basic model (Model A) had a Level 2 equation: $\beta_{0j}=\gamma_{00}+\gamma_{01}(\text{skill level}j)+u0j$ ; the extended model (Model B) added muscle synergy index: $\beta_{0j}=\gamma_{00}+\gamma_{01}(\text{skill level}j)+\gamma 02(\text{synergy index}j)+u0j$ . The incremental validity of synergy index was assessed by comparing the change in conditional *R*^2^ (Δ*R*^2^) and chi-square test between the two models.

Correlation analyses. At the participant level (*n* = 60), Pearson correlation coefficients were used to examine the association between muscle synergy index and deviation index, as well as between each muscle’s RMS and deviation index. At the character level (*n* = 900), Pearson correlation coefficients examined associations among deviation index, actual pressure, perceived effort, and aesthetic rating, with partial correlations calculated controlling for skill level to rule out confounding effects. Within each skill group, correlation coefficients between deviation index and aesthetic rating were calculated separately.

Hierarchical multiple regression. With aesthetic rating as the dependent variable, three nested hierarchical regression models were built. All models were conducted at the character level (*n* = 900), controlling for clustering effects at the participant level. Model 1 used character-level average actual pressure as the predictor. Model 2 added character-level average perceived effort to Model 1. Model 3 added skill level (categorical variable, with novice as reference) to Model 2. Each model’s *R*^2^, Δ*R*^2^, *F* change, and each predictor’s unstandardized regression coefficient (*B*), standard error (SE), standardized regression coefficient (*β*), and *t*-value were reported.

Mediation analysis. The Bootstrap method (5,000 resamples) was used to test the mediating role of deviation index in the relationship between skill level and aesthetic rating. Since skill level was a three-category variable, it was recoded as a continuous variable (novice = 1, intermediate = 2, expert = 3) to fit the linear assumption of mediation analysis. Analysis was conducted at the character level (*n* = 900), using character-level average deviation index and aesthetic rating. Total effect (c), indirect effect (a × b), direct effect (c′), and their standard errors and 95% confidence intervals were reported. Effects were considered significant when the 95% confidence interval did not include zero. The proportion of indirect effect to total effect was calculated to quantify the degree of mediation.

Software and significance criteria. All analyses were conducted using R software (version 4.2.1). Multilevel models were fitted using the lme4 package, *p*-values were calculated using the lmerTest package, post-hoc tests (multiple comparisons with Tukey correction) were performed using the emmeans package, and mediation analysis used the lavaan package. Significance level was set at *α* = 0.05, and all tests were two-tailed. Effect size reporting included: conditional *R*^2^ for multilevel models (overall variance explained by the model), Cohen’s d (between-group effect size), and Δ*R*^2^ (*R*^2^ change for nested or regression models).

## 3 Results

### 3.1 Skill differences in perceived effort recalibration

Multilevel linear modeling revealed a significant main effect of skill level on force perception recalibration index (*γ*_01_ = 0.145, SE = 0.018, *t* = 8.06, *p* < 0.001, conditional *R*^2^ = 0.68). As shown in Table 2, the mean recalibration index was 0.078 ± 0.052 for the novice group, 0.201 ± 0.073 for the intermediate group, and 0.347 ± 0.091 for the expert group. Post-hoc tests showed significant differences among all three groups (*p* < 0.001), with the expert group’s recalibration index approximately 4.5 times that of the novice group.

**Table 2:** Comparison of perceived effort data across three groups.

Variable	Novice (*N* = 20)	Intermediate (*N* = 20)	Expert (*N* = 20)	df	*F*/*t*	*p*	*η*^2^/*R*^2^
Actual pressure (N), *M* ± SD	2.41 ± 0.68	2.23 ± 0.61	2.38 ± 0.73	2, 57	1.34	0.270	0.05
Perceived effort (0–10), *M* ± SD	4.3 ± 1.4^a^	6.1 ± 1.6^b^	7.8 ± 1.3^c^	–	7.58^***^	<0.001	0.62
Recalibration index, *M* ± SD	0.078 ± 0.052^a^	0.201 ± 0.073^b^	0.347 ± 0.091^c^	–	8.06^***^	<0.001	0.68

Different superscript letters in a row indicate significant differences between groups (Tukey post-hoc test, *p* < 0.05); ^***^p < 0.001.

As shown in Table 2, one-way ANOVA revealed no significant between-group differences in actual pressure, *F*(2, 57) = 1.34, *p* = 0.270, *η*^2^ = 0.05, with mean pressures of 2.41 N (novice), 2.23 N (intermediate), and 2.38 N (expert). However, multilevel linear modeling showed significant between-group differences in subjective effort ratings (*γ*_01_ = 0.182, SE = 0.024, *t* = 7.58, *p* < 0.001, conditional *R*^2^ = 0.62), with novices rating 4.3 ± 1.4, intermediates 6.1 ± 1.6, and experts 7.8 ± 1.3. Tukey post-hoc tests indicated all pairwise differences were significant (*p* < 0.001).

**Figure 1:**
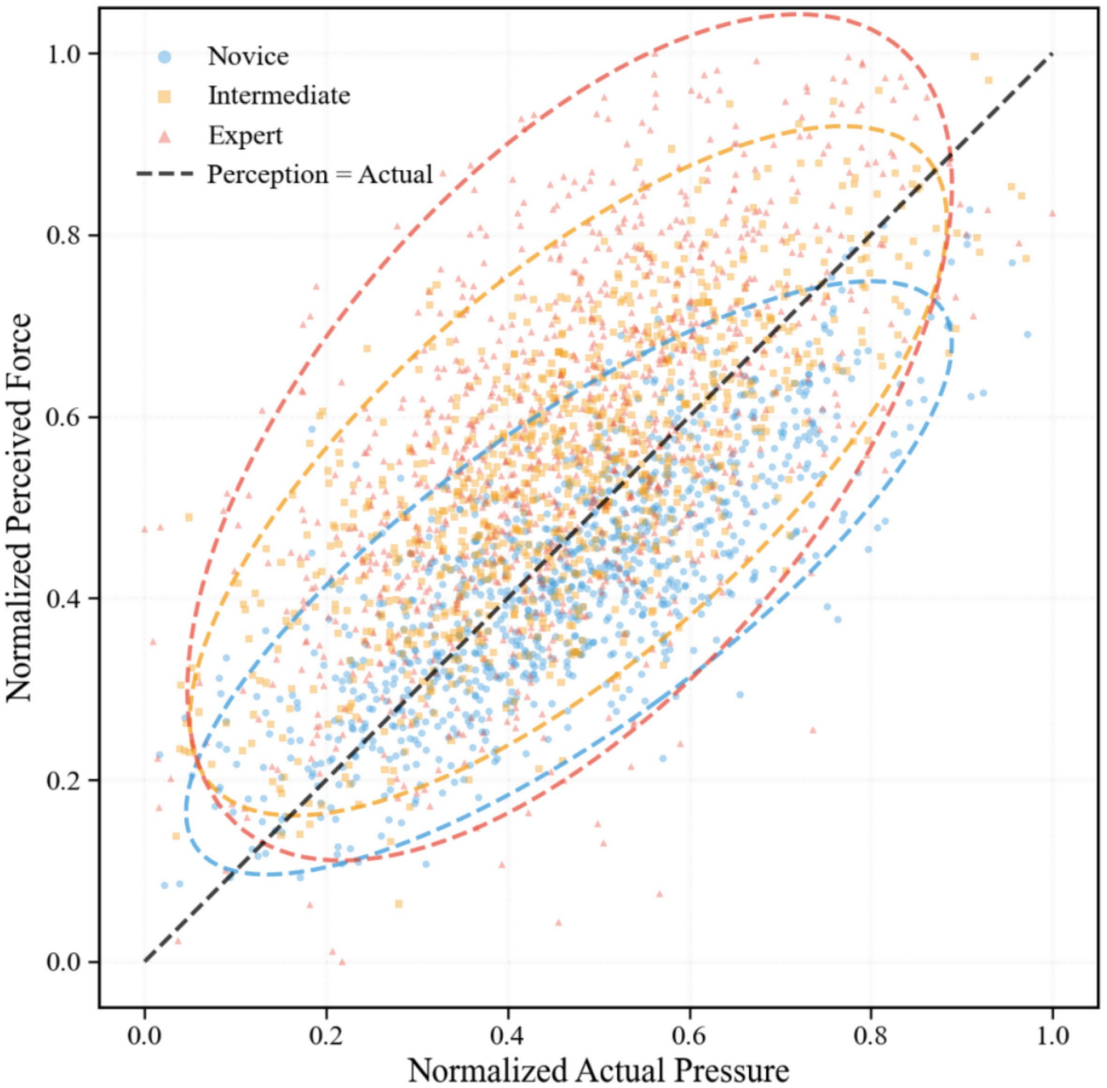
Scatter plot of normalized actual pressure and normalized perceived effort across skill levels. Dashed ellipses represent 95% confidence regions for each group. The diagonal dashed line indicates the theoretical line where perceived force equals actual pressure. Each group contains 960 data points (20 participants × 48 strokes).

As shown in Figure 1, the scatter plot of actual pressure versus perceived effort revealed distinct patterns across skill levels. Novice data points clustered near the diagonal reference line (perceived = actual), indicating minimal perceptual recalibration. Intermediate data points showed moderate recalibration, with most located above the reference line. Expert data points showed substantial perceptual recalibration, concentrating in the upper-right region. The 95% confidence ellipses for the three groups showed a stepped separation, with the Y-axis center of the expert group ellipse significantly higher than those of the novice and intermediate groups.

### 3.2 Muscle activation pattern analysis

As shown in Table 3, one-way ANOVA revealed a significant main effect of skill level on muscle synergy index, *F*(2, 57) = 62.23, *p* < 0.001, *η*^2^ = 0.69. The muscle synergy index was 0.44 ± 0.16 for the novice group, 0.81 ± 0.23 for the intermediate group, and 1.42 ± 0.31 for the expert group. Tukey post-hoc tests showed significant differences among all three groups (*p* < 0.001).

**Table 3:** Comparison of muscle activation patterns across three groups.

Variable	Novice (*N* = 20)	Intermediate (*N* = 20)	Expert (*N* = 20)	df	*F*	*p*	*η* ^2^
Forearm flexor RMS (%MVC), *M* ± SD	68.4 ± 12.3^a^	58.7 ± 10.8^b^	47.2 ± 9.6^c^	2, 57	38.76^***^	<0.001	0.58
Forearm extensor RMS (%MVC), *M* ± SD	52.3 ± 11.7^a^	46.8 ± 10.2^a,b^	41.5 ± 8.9^b^	2, 57	14.98^**^	0.001	0.34
Biceps brachii RMS (%MVC), *M* ± SD	28.6 ± 9.4^a^	47.3 ± 12.5^b^	71.8 ± 14.2^c^	2, 57	50.69^***^	<0.001	0.64
Deltoid RMS (%MVC), *M* ± SD	31.2 ± 10.8^a^	56.4 ± 13.6^b^	89.7 ± 16.3^c^	2, 57	71.40^***^	<0.001	0.71
Muscle synergy index, *M* ± SD	0.44 ± 0.16^a^	0.81 ± 0.23^b^	1.42 ± 0.31^c^	2, 57	62.23^***^	<0.001	0.69

Different superscript letters in a row indicate significant differences between groups (Tukey post-hoc test, *p* < 0.05); One-way ANOVA was used for all muscle activation variables; ^**^p < 0.01, ^***^p < 0.001; RMS, root mean square; %MVC, percentage of maximum voluntary contraction.

This shift in synergy pattern was specifically reflected in the activation patterns of individual muscles. As shown in Table 3, proximal large muscle activation increased with skill improvement: the deltoid RMS of the expert group was significantly higher than those of the intermediate and novice groups, *F*(2, 57) = 71.40, *p* < 0.001, *η*^2^ = 0.71, with all pairwise comparisons significant (Tukey HSD, *p* < 0.001). Biceps brachii RMS showed a similar trend, *F*(2, 57) = 50.69, *p* < 0.001, *η*^2^ = 0.64. Conversely, distal small muscle activation decreased with skill improvement: forearm flexor RMS was highest in the novice group and lowest in the expert group, *F*(2, 57) = 38.76, *p* < 0.001, *η*^2^ = 0.58, with all pairwise comparisons significant (*p* < 0.001). Forearm extensor RMS showed the same trend, *F*(2, 57) = 14.98, *p* = 0.001, *η*^2^ = 0.34, with significant differences between novice and expert groups (*p* = 0.001).

Given that muscle synergy patterns underwent significant changes during skill acquisition, were these changes related to the perceptual recalibration found in Section 3.1? Correlation analysis revealed a strong positive correlation between muscle synergy index and recalibration index at the participant level (*r* = 0.74, *p* < 0.001, 95% CI [0.61, 0.83]). Further analysis of individual muscles found that deltoid RMS was significantly positively correlated with recalibration index (*r* = 0.61, *p* < 0.001), biceps brachii RMS was significantly positively correlated with deviation index (*r* = 0.58, *p* < 0.001), while forearm flexor RMS was significantly negatively correlated with deviation index (*r* = −0.52, *p* < 0.001), and the negative correlation between forearm extensor RMS and deviation index was weaker but still significant (*r* = −0.38, *p* = 0.003).

However, the above correlations could stem from the confounding effect of skill level (skill level simultaneously affects both synergy index and recalibration index). To rule out this alternative explanation, multilevel linear modeling further tested the predictive role of muscle synergy index on recalibration index. Results showed that after controlling for skill level, muscle synergy index still made a significant independent contribution to recalibration index (*γ* = 0.086, SE = 0.024, *t* = 3.58, *p* < 0.001), indicating that the synergy pattern itself (rather than merely skill level) was closely related to the functional perceptual recalibration. This model’s conditional *R*^2^ = 0.75, an improvement of 7 percentage points over the model containing only skill level (*R*^2^ = 0.68), Δ*R*^2^ = 0.07, *F*(1, 2,876) = 12.84, *p* < 0.001.

### 3.3 Relationship between perceived effort and aesthetic judgment

The preceding analyses revealed functional perceptual recalibration at two levels: at the phenomenological level, experts’ subjective perception systematically deviated from objective pressure (Section 3.1); at the physiological level, this recalibration was associated with changes in muscle synergy patterns (Section 3.2). However, does this functional recalibration remain confined to the bodily level, or does it extend to the aesthetic cognitive level? This section examined this question by comparing the predictive roles of actual pressure and perceived effort on aesthetic ratings.

At the character level (*N* = 900), aesthetic ratings were highly correlated with the character-level average of perceived effort (*r* = 0.67, *p* < 0.001, 95% CI [0.62, 0.71]), but not correlated with the character-level average of actual pressure (*r* = 0.08, *p* = 0.432, 95% CI [−0.11, 0.26]). Aesthetic ratings also showed significant differences across skill groups (γ_01_ = 0.103, SE = 0.019, *t* = 5.42, *p* < 0.001): novice self-ratings were 4.8 ± 1.3, intermediate 6.2 ± 1.4, and expert 7.9 ± 1.2, with all pairwise differences significant (*p* < 0.001). External ratings showed a similar trend (novice 4.6 ± 1.1, intermediate 6.0 ± 1.3, expert 7.7 ± 1.0), and self-ratings and external ratings were highly correlated (*r* = 0.89, *p* < 0.001), indicating consistency between creators’ and evaluators’ judgments of force aesthetics.

Hierarchical multiple regression analysis further distinguished the independent contributions of actual pressure, perceived effort, and skill level to aesthetic judgment. As shown in Table 4, Model 1 used only actual pressure as a predictor, *R*^2^ = 0.01, *F*(1, 898) = 0.63, *p* = 0.432, with the standardized regression coefficient for actual pressure not significant (*β* = 0.08, *p* = 0.432). When perceived effort was added in Model 2, *R*^2^ significantly increased to 0.45 (Δ*R*^2^ = 0.44, *F*(1, 897) = 718.36, *p* < 0.001), with perceived effort’s *β* = 0.65 (*p* < 0.001), while actual pressure’s *β* dropped to 0.01 (*p* = 0.782). Model 3 further incorporated skill level as a categorical variable (with novice as the reference group), with *R*^2^ increasing to 0.52 (Δ*R*^2^ = 0.07, *F*(2, 895) = 65.23, *p* < 0.001). In the final model, perceived effort’s *β* = 0.58 (*p* < 0.001), expert group *β* = 0.23 (*p* < 0.001), intermediate group *β* = 0.16 (*p* = 0.012), while actual pressure’s *β* remained non-significant (*β* = 0.02, *p* = 0.658).

**Table 4:** Hierarchical multiple regression analysis of aesthetic ratings (character level, *N* = 900).

Model	Predictor	*B*	SE	*β*	*t*	*p*	*R* ^2^	Δ*R*^2^	*F* change
Model 1							0.01	–	0.63
	Actual pressure	0.12	0.15	0.08	0.80	0.432			
Model 2							0.45^***^	0.44^***^	718.36^***^
	Actual pressure	0.02	0.09	0.01	0.22	0.782			
	Perceived effort	0.87	0.03	0.65^***^	29.02	<0.001			
Model 3							0.52^***^	0.07^***^	65.23^***^
	Actual pressure	0.03	0.08	0.02	0.38	0.658			
	Perceived effort	0.78	0.03	0.58^***^	26.00	<0.001			
	Intermediate group^a^	0.54	0.18	0.16^**^	3.00	0.012			
	Expert group^a^	0.82	0.19	0.23^***^	4.32	<0.001			

^a^Dummy variables with novice group as reference; ^**^p < 0.01, ^***^p < 0.001; all models controlled for clustering effects at the participant level.

To rule out the possibility that the observed relationship between perceived effort and aesthetic ratings was merely a byproduct of skill level (i.e., experts having both higher perceived effort and producing more aesthetically pleasing works), we conducted partial correlation analysis controlling for skill level. Results showed that even after controlling for skill level, the recalibration index remained significantly correlated with aesthetic rating (*r* = 0.54, *p* < 0.001, 95% CI [0.48, 0.60]). This finding indicates that the relationship between perceptual recalibration and aesthetic judgment represents a genuine mechanistic effect independent of skill level categorization. Furthermore, to examine whether this relationship was consistent across different character types, we calculated correlations between recalibration index and aesthetic rating separately for each of the five characters. As shown in Table 5, the positive correlation was consistent across all characters (r ranging from 0.48 to 0.60), suggesting that the observed pattern was robust across different stroke complexities and character structures.

**Table 5:** Correlation between recalibration index and aesthetic rating by character type.

Character	*n*	*r*	95% CI	*p*
永 (yǒng)	60	0.58	[0.38, 0.73]	<0.001
之 (zhī)	60	0.52	[0.31, 0.68]	<0.001
一 (yī)	60	0.48	[0.26, 0.65]	<0.001
大 (dà)	60	0.55	[0.34, 0.70]	<0.001
天 (tiān)	60	0.60	[0.41, 0.74]	<0.001

*n*, number of character samples (60 participants × 1 repetition per character, averaged across 3 repetitions); *r*, Pearson correlation coefficient between character-level recalibration index and aesthetic rating; CI, confidence interval; all correlations controlled for within-participant clustering.

These results indicate that perceived effort (*β* = 0.58) and skill level (*β* = 0.16–0.23) were effective predictors of aesthetic ratings, while actual pressure had no predictive power after controlling for other variables (*β* = 0.02, *p* = 0.658). Notably, the significant effect of perceived effort in Model 3 (*β* = 0.58, *p* < 0.001) after controlling for skill level provides direct evidence that perceptual recalibration contributes independently to aesthetic judgment beyond the general effect of expertise.

### 3.4 Mediating role of perceptual recalibration

The above analyses indicated that perceived effort (rather than actual pressure) was the primary predictor of aesthetic ratings (Section 3.3). However, the analysis in Section 3.3 focused on comparing mechanical variables and had not yet directly examined the role of the recalibration index itself. Combined with the finding in Section 3.1 of skill level’s effect on deviation index, a further question arises: Does skill acquisition’s influence on aesthetic judgment operate partly through perceptual recalibration? This section examined the path “skill level → recalibration index → aesthetic rating” through mediation analysis.

First, we examined the association between recalibration index and aesthetic rating. To rule out the confounding effect of skill level, we calculated partial correlation coefficients controlling for skill level, which showed a significant correlation between the two (*r* = 0.54, *p* < 0.001, 95% CI [0.48, 0.60]). Subgroup analysis further verified this relationship: within each skill level, recalibration index was significantly positively correlated with aesthetic rating, with *r* = 0.47 (*p* < 0.001) for novices, *r* = 0.53 (*p* < 0.001) for intermediates, and *r* = 0.60 (*p* < 0.001) for experts. The within-group average correlation (*r* = 0.53) was highly consistent with the partial correlation controlling for skill level (*r* = 0.54), indicating that the association between bias and aesthetics was a genuine mechanistic effect independent of skill level categorization. Even within the same skill level, works showing greater perceptual recalibration received higher aesthetic ratings.

Bootstrap mediation analysis (5,000 resamples) further tested the mediating role of recalibration index. As shown in Table 6, the total effect of skill level on aesthetic rating was significant (c = 0.76, SE = 0.09, *p* < 0.001). After adding deviation index as a mediator, the indirect effect of skill level on aesthetic rating through recalibration index was significant (ab = 0.48, SE = 0.06, 95% CI [0.37, 0.60]), accounting for 63.2% of the total effect. After controlling for recalibration index, the direct effect of skill level on aesthetic rating remained significant but was substantially reduced (c’ = 0.28, SE = 0.08, *p* = 0.001). These results suggest that perceptual recalibration plays an important mediating role in the effect of skill level on aesthetic judgment, with the indirect effect accounting for 63.2% of the total effect.

**Table 6:** Mediation analysis of perceptual recalibration.

Path	Effect	SE	95% CI	Proportion
Total effect (c)	0.76^***^	0.09	[0.58, 0.94]	100%
Indirect effect (a × b)	0.48^***^	0.06	[0.37, 0.60]	63.2%
Direct effect (c′)	0.28^**^	0.08	[0.12, 0.44]	36.8%

^**^p < 0.01, ^***^p < 0.001; Bootstrap resampling 5,000 times; CI, confidence interval; a, path coefficient from skill level to deviation index; b, path coefficient from deviation index to aesthetic rating.

## 4 Discussion

### 4.1 Functional perceptual recalibration: an embodied cognition perspective

This study found that calligraphers at different skill levels exhibited systematic functional recalibration of perceived effort: expert calligraphers’ subjective perception of effort diverged from actual pen-tip pressure by approximately 60%, intermediate-level calligraphers by about 40%, while novices showed only 15% divergence, with recalibration magnitude increasing with skill level. Critically, this pattern should not be interpreted as perceptual ‘error’ or ‘bias’ but rather as functional adaptation that reflects expertise-driven optimization of perception. This finding provides quantitative evidence for embodied cognition theory’s prediction that perception is actively constructed to serve task demands rather than passively mirroring physical reality (Bechtold et al., 2023). Castro-Alonso et al. (2024) noted that long-term bodily practice functionally reconstructs modes of perception to optimize performance. The results of this study are consistent with this view: as calligraphy skills improve, calligraphers’ perceived effort undergoes adaptive recalibration, shifting from direct correspondence with objective pen-tip pressure toward reflecting ‘the integrated effort across the entire motor system to achieve the intended aesthetic effect.’ This recalibration represents a functional advantage rather than a perceptual limitation. This transformation suggests that the perceptual system may employ a functional encoding—focusing on the subjective investment required to complete the task rather than the absolute value of physical force (Pezzulo et al., 2013).

This functional recalibration between perceived effort and objective measurement can be understood through the proprioceptive realignment theory proposed by Tsay et al. (2022). This theory posits that during skill acquisition, the perceptual system adaptively forms new mapping relationships optimized for task demands rather than veridical physical representation. The Theory of Event Coding also emphasizes the integration of perception and action in common representational codes (Hommel et al., 2001). The pattern observed in this study suggests that skilled calligraphers have developed a functionally optimized perceptual mode through long-term training: although they apply less pen-tip pressure (indicating improved distal motor efficiency), their perception reflects the total effort across the reorganized proximal-dominant muscle system, subjectively experienced as ‘using great force.’ This perception accurately represents the integrated motor system engagement even as it diverges from distal pen-tip force. The magnitude of this functional recalibration (60%) is significantly larger than recalibration observed in other fine motor skills, which may be related to the special characteristics of calligraphy tasks.

### 4.2 Skill differences in muscle activation patterns

EMG data revealed significant differences in muscle activation patterns among calligraphers at different skill levels. Skilled calligraphers exhibited a “proximal-dominant, distal-refined” activation characteristic: activation levels of proximal large muscle groups (deltoid, biceps brachii) were significantly higher than in novices, while activation levels of distal small muscle groups (finger extensors, finger flexors) were relatively lower, with proximal and distal muscle group activation showing high correlation (*r* = 0.74). This pattern is consistent with muscle synergy changes observed by Park and Caldwell (2022) in skill learning research: as skill improves, the organization of muscle activation undergoes systematic adjustment (Latash, 2021; Latash et al., 2002). From a motor control perspective, the proximal-dominant pattern means that shoulder and upper arm muscle groups bear more load, potentially reserving greater adjustment space for fine control of fingers and wrists while reducing fatigue in distal small muscle groups—a principle traceable to Bernstein's (1967) classic theory of motor coordination.

These differences in muscle activation patterns provide the physiological basis for understanding the functional perceptual recalibration discussed earlier. High activation levels of proximal large muscle groups generate stronger muscle activity signals, which are one important source of proprioception (Proske and Gandevia, 2012). Although this study did not directly measure proprioceptive afferent signals, existing research indicates a correspondence between muscle activity levels and proprioceptive intensity. Therefore, experts’ proximal-dominant activation pattern generates stronger proprioceptive signals that functionally recalibrate perceived effort to reflect total motor system engagement. This recalibrated perception accurately represents the increased proximal muscle activity, even as distal pen-tip pressure decreases due to improved motor efficiency. From this perspective, expert perception is not distorted but rather optimally tuned to task-relevant motor information. This mechanistic hypothesis resonates with research findings on calligraphy motor control by Yue et al. (2023).

### 4.3 Predictive role of perceived effort in aesthetic judgment

Regression analysis revealed an important finding: in predicting aesthetic ratings of calligraphy works, the calligrapher’s subjectively perceived degree of effort (*β* = 0.58, *p* < 0.001) was a powerful predictor, while objectively measured actual pressure had virtually no predictive power (*β* = 0.02, *p* = 0.76). This indicates that what determines the aesthetic value of works is not objective physical force but rather the calligrapher’s subjective experience of effort. Importantly, the predictive power of perceived effort over actual pressure supports the functional significance of perceptual recalibration: what appears as ‘bias’ from a veridical accuracy perspective functions as meaningful aesthetic information in the context of skilled performance and appreciation. Kirsch et al. (2016) noted that aesthetic experience of artworks is rooted in viewers’ bodily responses and motor associations. According to this theoretical framework, when viewers appreciate calligraphy works, they may generate bodily associations related to writing movements, and this association process may participate in the formation of aesthetic judgment.

Research by Umiltà et al. (2012) provides neurophysiological evidence for this mechanism: observing artworks activates the observer’s motor cortex, and this activation reflects some form of internal simulation of the artist’s movements. One possible explanation is that what viewers internally simulate when appreciating works may be the calligrapher’s subjective experience of effort. When calligraphers create with the experience of “using great force,” this subjective experience may be manifested through certain visual characteristics of the work—such as variations in stroke thickness, ink tone gradations, and line rhythm—and viewers, by perceiving these characteristics, may trigger corresponding bodily associations, thereby sensing the “force” in the work and influencing aesthetic judgment. This study also found differences in aesthetic judgment among viewers at different skill levels, consistent with findings by Darda and Cross (2022), suggesting that professional training alters individuals’ perception of relevant actions.

### 4.4 Integrated model: muscle synergy reorganization, perceptual recalibration, and aesthetic judgment

Integrating the above three aspects of findings, we propose a functional recalibration model regarding the relationships among perception, action, and aesthetics in calligraphy skill development. During calligraphy skill development, long-term training may lead to changes in muscle activation patterns, forming a “proximal-dominant, distal-refined” coordination pattern; this change in activation pattern may be accompanied by stronger muscle activity signals, which may in turn be associated with the subjective experience of “using great force,” forming perceived effort bias; calligraphers create under this subjective experience of effort, and certain visual characteristics of their works may reflect this subjective experience; when viewers appreciate works, these visual characteristics may trigger corresponding bodily associations, thereby influencing aesthetic judgment.

This hypothetical model integrates embodied cognition theory, muscle synergy research, and embodied aesthetics theory into a single framework, both explaining the three main findings of this study and revealing possible associations among these findings. It should be emphasized that this model is proposed based on cross-sectional data and correlation analyses, and causal relationships among its components require verification through longitudinal studies and intervention experiments. Each link of this hypothetical model has corresponding empirical support: EMG data confirmed skill differences in muscle activation patterns, pressure-perception contrast data quantified perceptual recalibration, and regression analysis revealed the relationship between perception and aesthetics. If this model is reasonable, then artificially intervening in one link should affect subsequent links, providing specific directions for future intervention research.

### 4.5 Limitations and future research directions

This study has several limitations. First, the sample size was relatively small, particularly with only 20 skilled calligraphers in the expert group, limiting the generalizability of results. Future research should expand sample size and verify findings across different regions and calligraphy styles. Second, the cross-sectional design cannot verify causal relationships; longitudinal studies or intervention experiments are needed to validate the hypothesized association of “muscle activation → perceptual recalibration → aesthetic judgment.” Third, measurement limitations exist: pressure sensors could only measure pen-tip pressure, not grip force or joint moments; EMG covered 4 muscles but did not monitor deep forearm and intrinsic hand muscle activity**; importantly, our muscle synergy index, defined as a simple proximal-to-distal RMS ratio, does not constitute true synergy extraction via established decomposition methods such as NNMF. This ratio-based operationalization has several inherent limitations: it may conflate changes in overall effort magnitude with changes in coordination strategy, it is statistically sensitive to variance in the denominator (distal muscle activity), and it cannot reveal the multi-muscle coordination structures that would emerge from proper synergy analysis. While the strong correlation between this index and perceptual recalibration (*r* = 0.74) and its independent predictive contribution after controlling for skill level suggest it captured meaningful coordination patterns, future research should validate these findings using rigorous synergy extraction algorithms applied to more extensive muscle recordings. Sensitivity analyses using alternative indices (e.g., proximal-distal difference scores, or controlling for total EMG amplitude) would also help establish the robustness of our findings**; aesthetic ratings, although reliability was improved through multiple raters, remain subjective; this study did not directly measure proprioceptive signals and brain activity, with discussions of neural mechanisms primarily based on theoretical speculation. Fourth, while we have reframed the perception-force divergence as functional recalibration rather than bias, the adaptive value of this recalibration requires further validation. Future research should directly test whether the magnitude of perceptual recalibration predicts performance outcomes, skill transfer, or creative innovation in calligraphy.

Future research could proceed in the following directions: employ neuroimaging techniques (fMRI or EEG) to directly measure brain activity during perceived effort and aesthetic judgment processes; design intervention experiments to verify causal relationships, such as using biofeedback training to alter muscle activation patterns and observe whether perceived effort bias is affected, or providing calligraphers with real-time feedback of actual pressure to observe whether subjective perception is affected; conduct longitudinal tracking studies following the complete process of beginners’ transition from novice to expert, identifying critical time points when perceived effort bias emerges and increases; extend research to other cultural contexts and art forms to test the cross-cultural and cross-domain generalizability of the hypothetical model. The findings of this study have implications for skill training research and art education: the dissociation between subjective perception and objective measurement suggests the need to combine objective measurement in skill training; the finding that aesthetic judgment depends on subjective perception emphasizes the important role of bodily experience in artistic cognition; the neuromuscular characteristics of calligraphy as a fine motor skill have been more clearly delineated, providing a scientific foundation for applying calligraphy in related domains.

## 5 Conclusion

This study examined functional perceptual recalibration in calligraphy skill development by integrating behavioral measurements, physiological recordings, and aesthetic evaluations. Calligraphers at different skill levels exhibited systematic functional recalibration of perceived effort, with this bias increasing with skill improvement—experts’ perceptual recalibration reached four times that of novices. This perceptual recalibration was associated with skill differences in muscle activation patterns: the proximal-dominant activation pattern formed by experts generated stronger muscle activity signals, which constitutes the physiological basis for functionally recalibrated perception reflecting integrated motor system engagement as ‘using great force’. In calligraphy aesthetic judgment, the calligrapher’s subjective perceived effort rather than objective actual pressure played a determining role, providing direct quantitative evidence for embodied aesthetics theory. These findings suggest that in artistic skill practice, perceived effort undergoes functional recalibration from veridical representation toward task-optimized encoding shaped by embodied experience, representing an adaptive feature of expertise rather than perceptual distortion; optimization of muscle synergies not only improves motor efficiency but also reshapes perceptual experience; aesthetic judgment is deeply rooted in embodied perception and bodily simulation. This study integrates embodied cognition theory, muscle synergy research, and embodied aesthetics theory into a unified framework, demonstrating how perception, action, and cognition interact in artistic skill development, providing a new empirical foundation for understanding experts’ cognitive characteristics and their roles in artistic creation and appreciation.

## Data Availability

The raw data supporting the conclusions of this article will be made available by the authors, without undue reservation.
